# Empowerment of the older adults in the context of Chinese culture: an evolutionary concept analysis

**DOI:** 10.3389/fpsyg.2023.1271315

**Published:** 2023-11-09

**Authors:** Shibo Zhang, Junfeng Li, Jingjie Zou, Yating Ai, Siqi Qin, Xixi Xiao, Hui Hu, Yuncui Wang

**Affiliations:** ^1^School of Nursing, Hubei University of Chinese Medicine, Wuhan, China; ^2^Department of Obstetrics and Gynecology, Women and Children’s Hospital of Chongqing Medical University (CQMU-WCH), Chongqing, China

**Keywords:** older people, empowerment, Chinese culture, conceptual analysis, collectivism

## Abstract

**Background:**

With the increasing focus on addressing the challenges of aging, researchers have begun to recognize the potential impact of empowering older individuals in addressing retirement issues. However, within different cultural contexts, there still needs to be a more precise analysis regarding the definition of empowering older individuals.

**Objective:**

To define and analyze the concept of empowering older individuals within the cultural context of China.

**Method:**

Using Rodgers’ concept analysis approach, a search was conducted in five databases (PubMed, Web of Science, China National Knowledge Infrastructure, Wan fang Data, and VIP Chinese Journal Platform) for studies on empowering older individuals from the time the databases were established until February 2023. The main disciplines involved in the search included nursing, medicine, and public health.

**Results:**

Out of the 7,028 studies, 50 articles met the inclusion criteria. The identified attributes are as follows: support system, belief change, and behavioral autonomy. The antecedents were grouped into four categories: physical obstacles, psychological concerns, personal needs and external challenges. The consequences were determined to be improved quality of life, reduced burden of old-age care, gain respect, and self-actualization.

**Conclusion:**

Empowering older individuals is a dynamic and evolving concept that involves aligning personal aspirations with appropriate external resources and expressing a certain degree of belief and behavioral change. This study deepens our understanding of empowering older individuals through comprehensive concept analysis, and the identified attributes, antecedents, and consequences of empowering older individuals can be utilized in practice, education, and research.

## 1. Introduction

Currently, countries worldwide strive to reduce the cost of elderly care, provide high-quality healthcare services, and improve the overall experiences of older individuals. However, older individuals face health challenges and a decline in their quality of life due to aging. They are eager to find measures to improve their well-being in areas such as employment, health, learning, and social participation (Fang et al., 2020) to enhance their overall quality of life. In this context, adopting evidence-based, innovative service approaches becomes crucial in supporting older individuals. Empowering older individuals involves helping them gain control over factors that impact their lives and improve their overall experiences (Gibson, 1991). However, it is essential to note that the abuse of power can also have negative consequences.

Considering the principles of person-centered treatment and the changing needs of older people, healthcare providers and social worker should proactively adapt to the emerging demands. Eliminating inequalities within relationships with older adults is of paramount importance (Zhang and Jiang, 2021).

Empowering older adults involves a wide range of conditions and services to ensure their autonomy and participation in various aspects of life, including healthcare, social engagement, and family dynamics while protecting their legal rights. The concept of empowerment originated from the civil rights movement and women’s liberation movement in the 1960s, gradually extending to other marginalized groups. Since the 1980s, it has been applied across diverse fields, such as public health, welfare, and mental well-being, leading to the development of numerous empowerment methods and definitions for different populations (Segal et al., 1995).

With the increasing use and application of empowerment, the methods and elements employed in empowering older adults have undergone significant changes. One widely used definition worldwide is described by Wallerstein and Bernstein (1988) as “a social action process that promotes the participation of individuals, organizations, and communities in controlling their lives within their communities and in the broader society.” Rodwell (1996) emphasizes the issues of partnership, equal decision-making, and freedom of choice in empowering older adults. Furthermore, Shearer (2009) interprets empowerment from the perspective of the empowered individual as “a purposeful process of engaging in personal and environmental change, recognizing patterns, and mobilizing internal resources to achieve well-being.” Some researchers (Casado et al., 2020) introduced empowerment as a means to help older adults gain control over negative factors impacting their lives. Moreover, a literature review (Higgins et al., 2017) suggests that in many texts, terms, and concepts such as “rights, participation and involvement, engagement, support, activation, collaboration” are used interchangeably or overlap in meaning.

Inaccurate naming and misuse of concepts can pose barriers to achieving optimal and ideal empowerment, thereby affecting the implementation and evaluation of empowerment efforts. A correct understanding of empowerment for older adults not only specifies the role of the empowerment provider but also lays the foundation for designing well-structured empowerment programs. Rodgers (2000) states that a concept evolves over time and undergoes changes influenced by cultural, social, disciplinary, temporal, and shared theoretical factors. Therefore, this study aims to reconstruct the concept of empowerment for older adults in contemporary Chinese culture. This is achieved by conducting a review of pertinent research on the empowerment of older adults in China, extracting and organizing its various applications, including antecedents, attributes, and consequences. This provides a basis for developing rigorous assessment tools and is a reference for subsequent screening, intervention research, and practices. We hope this work can contribute to promoting older adults’ health, well-being, and social development.

## 2. Materials and methods

### 2.1. Aim

A proper understanding of the empowerment of older adults from a cultural perspective can assist researchers in that culture in designing empowerment processes that align more closely with their cultural context. Additionally, it can provide a foundation for researchers in other cultures to engage in cross-cultural research. This paper aims to employ Rodgers’ conceptual analysis approach to examine the evolving concept of empowering older adults in China, a culture known for its collectivist values, and to serve as a basis for further research by other scholars in the future.

### 2.2. Design

Concept analysis helps to determine the shared meaning of a concept and lays the foundation for subsequent education, practice, and theoretical development (Foronda, 2008). This study will use Rodgers’ concept analysis method for this review (Table 1). There are two reasons for this choice. Firstly, Rodgers’ concept analysis method is a rigorous inductive approach, where researchers’ preconceptions do not influence the abstract analysis process. Secondly, Rodgers (2000) also acknowledges that a concept undergoes specific changes influenced by underlying factors such as culture, social groups, or time periods. This aligns with the approach of this study. Therefore, using Rodgers’ concept analysis method can explore the dynamic influence of culture on concepts. This study has been registered in PROSPERO (CRD42023427066). The Preferred Reporting Items for Systematic Reviews and Meta-Analyses (PRISMA) was applied in this study (Page et al., 2021; Supplementary File S1).

**Table 1:** Steps for Rodgers evolutionary concept analysis.

Step	Description
1	Identify a concept and its surrogate terms
2	Determine and select a suitable data collection scope
3	Data collection
	1.Concept attributes
	2.Contextual basis including temporal, sociocultural, and interdisciplinary variables
4	Data analysis
5	If necessary, provide examples supporting the concept
6	Identify hypotheses and applications for the concept’s future development

### 2.3. Search methods

**Figure 1:**
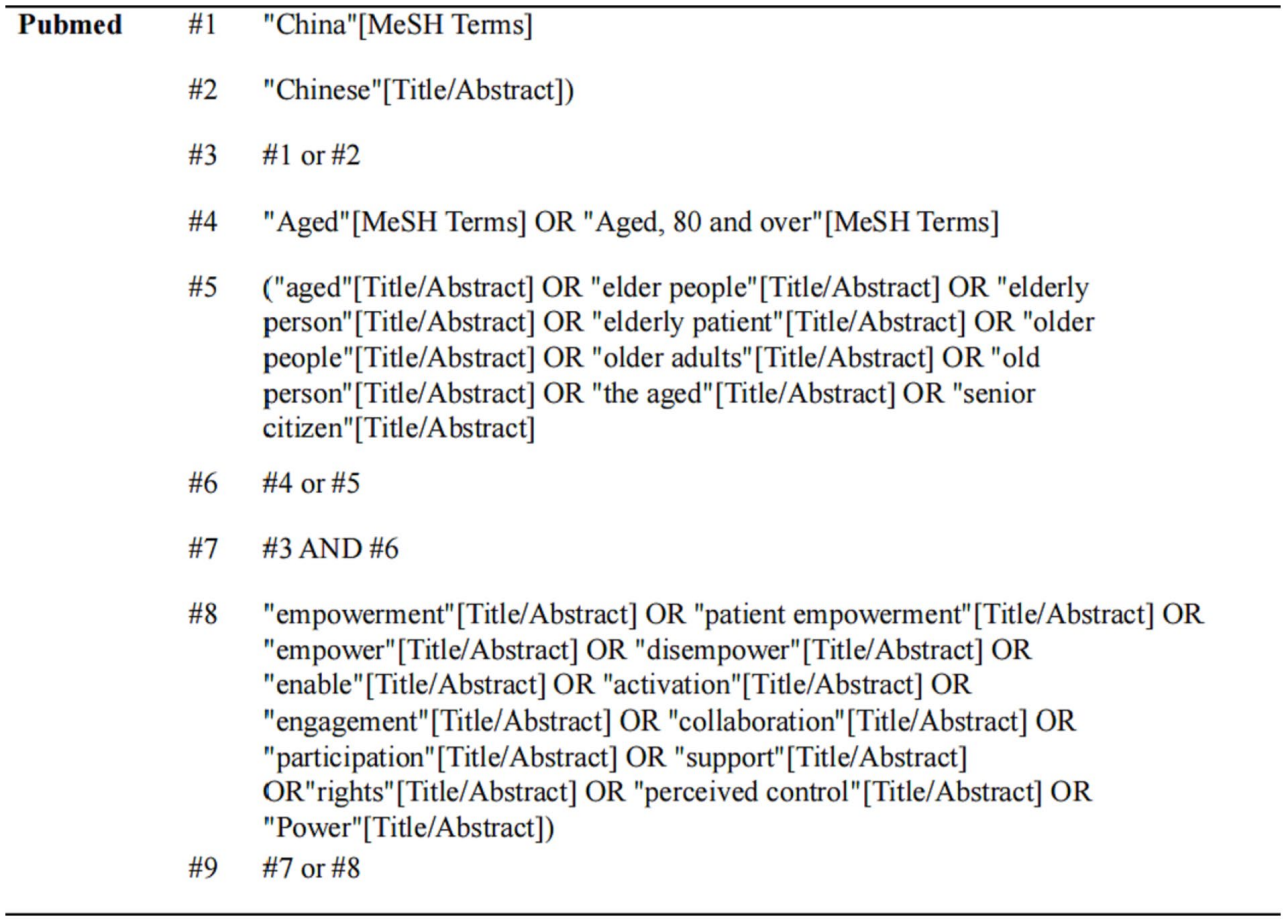
Search strategy in PubMed.

We selected a combination of five databases for literature search in concept analysis, including PubMed, Web of Science, China National Knowledge Infrastructure (CNKI), Wanfang Database, and VIP Database. Two researchers (SQ and XX) conducted the search in March 2023, covering data from these databases up until February 2023. We formulated search terms and utilized subject headings whenever possible, with necessary adjustments for the specific requirements of each database. Three groups of keywords or MeSH terms were included and combined using Boolean operators: (1) aged*, elder people*, elderly person*, elderly patient*, older people*, older adults*, old person*; (2) empowerment, patient empowerment*, empower*, disempower*, collaboration*, participation*, involvement*, support*, rights*, enable*, activation*, engagement*, perceived control*, Power*; (3) China*, Chinese*, Chinese people.” To determine the eligibility of potentially relevant studies, researchers reviewed all titles and abstracts. Figure 1 shows an example of the search strategy using PubMed. For specific search strategies, please refer to (Supplementary File S2).

#### 2.3.1. Inclusion and exclusion criteria

##### 2.3.1.1. Inclusion criteria

1. Eligible studies include original articles, review articles, and practice research.
2. The theme of this article is empowerment.
3. The research subjects are older adults (age ≥ 65 years).
4. This article is written in full-text in either Chinese or English.

##### 2.3.1.2. Exclusion criteria

1. The target population does not include Chinese individuals.
2. Studies that have not been published in peer-reviewed journals, case reports, conference proceedings and poster abstracts are excluded.
3. The study subjects consists of healthcare professionals, community workers, or family caregivers.

### 2.4. Search outcomes

**Figure 2:**
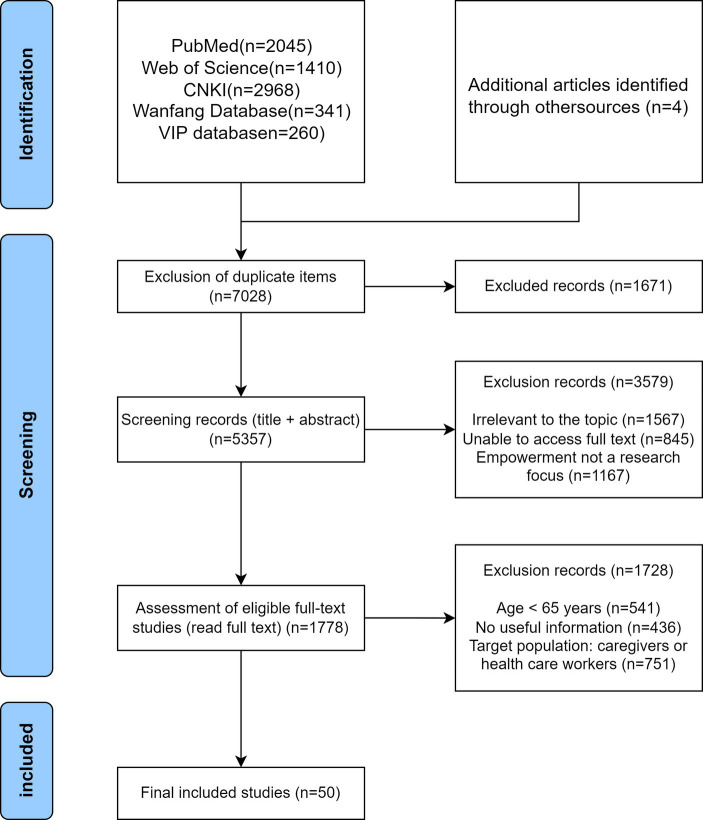
Flowchart of the selection process for the conceptual analysis study.

Three researchers independently screened and extracted literature according to the inclusion and exclusion criteria. The preliminary search using the aforementioned strategy yielded a total of 7,028 articles. In the first step, after excluding duplicate entries, there were 5,357 studies remaining. In the second step, after reviewing the titles and abstracts of the articles, those unrelated to the topic, inaccessible full texts, and articles where empowerment was not the focus were excluded, leaving 1778 articles. In the third step, after reading the full texts of the selected articles, articles were excluded if the age of the study participants was less than 65 years; not Chinese; no usable information; or the study subjects consisted of family caregivers or healthcare professionals for older adults. Finally, 50 articles remained. The search process is illustrated in Figure 2.

### 2.5. Data extraction and synthesis

Each article was coded based on antecedents, attributes, consequences. Well-trained graduate students and experienced professors participated in the analysis. The coding process used Rodgers’ evolutionary method. Five researchers independently extracted the data’s definitions and antecedents, attributes and consequences. First, we researched the concept to gain an initial impression and understanding of its nature and then had several rounds of discussion before content extraction and abstract extraction. We carefully read the data collection in the review system to ensure high-quality extraction and analysis, and next, we carefully read the complete papers to be analyzed for each. The comprehensive report of the article extracted the relevant parts of antecedents, attributes, and consequences from within the article, recorded them on the corresponding coding sheet, and then combined the same or similar contents to compare and classify all extracted contents until the conceptual function became clearer. Any disagreements among the five investigators were resolved through discussion, and together, they created a table of attributes, antecedents, and consequences.

## 3. Results

### 3.1. Study characteristics

This analysis includes studies published between the database establishment and February 2023. The included quantitative studies (*n* = 17), qualitative study designs (*n* = 16), mixed methods research (*n* = 11), and reviews (*n* = 6). The sample sizes varied widely, ranging from as low as 16 to as high as 812. The disciplines involved, differentiated by domain keywords, author backgrounds, and article topics, include nursing (*n* = 39), public health (*n* = 7), and medicine (*n* = 4; Supplementary File S3).

### 3.2. Attributes

The definition of attributes allows for identifying and differentiating a concept from others. Our analysis has determined the most common characteristics of empowered elderly individuals within Chinese culture. These can be summarized as follows: (1) Support system, (2) Belief change, and (3) Behavioral autonomy.

#### 3.2.1. Support system

##### 3.2.1.1. Resource support

Resource support emerges as the central theme in all reviewed literature, being mentioned in 26 articles. Multiple studies (Lambrinou et al., 2019; Vainauskienė and Vaitkienė, 2021) indicate that the higher the level of support older adults receive, the greater their level of empowerment. This is attributed to the fact that these resources provide older adults with increased choices and control, thereby enhancing their autonomy and sense of accomplishment. Shearer (2009) classifies these resources into three categories: personal resources, family resources, and social resources. Personal resources encompass individual abilities and potentials, including education, emotions, and interpersonal relationships (friends, partners), which help older adults enhance self-awareness, emotional stability, and foster positive relationships to adapt to declining physical conditions and changes in their living environment. Chinese studies particularly underscore the significance of family resources, such as relatives and family responsibilities, highlighting the importance of family support in empowering older adults. Social resources refer to the influence and support derived from the social networks in which older adults are engaged, encompassing support such as social services and informational materials. These resources can provide older adults with increased opportunities and platforms to enhance their social participation and decision-making.

##### 3.2.1.2. Knowledge and ability support

Several scholars (Camerini et al., 2012; Diviani et al., 2012) have conducted extensive research on the connection and differentiation between knowledge and empowerment, highlighting the significance of knowledge and skills in the process of empowerment. On one hand, possessing knowledge and skills enables older adults to independently navigate through various challenges in their everyday lives. On the other hand, when older adults have the knowledge and skills to solve problems, it ensures that their voices are heard and respected, both in their personal lives and in social and political matters. Therefore, scholars emphasize the importance of fostering self-efficacy (Beh and Ang, 2022), learning ability (Chang and Park, 2012), and information literacy (Zhao et al., 2022) among older adults. Additionally, some researchers (Geraniou and Jankvist, 2019) believe that knowledge and abilities serve as a bridge between beliefs and actions. Having sufficient knowledge and abilities can assist older adults in identifying areas for improvement, gaining a stronger sense of control over their lives, and establishing the foundation and prerequisites for implementing changes.

#### 3.2.2. Belief change

##### 3.2.2.1. Self-reflection

Self-reflection (Hage and Lorensen, 2005) refers to the process in which individuals deeply contemplate and analyze their goals, responsibilities, and values. The characteristics of self-reflection in older adults (Tsubouchi et al., 2021) involve strengthening self-awareness through self-examination, objective evaluation, subjective and objective issue perception, and the clarification of responsibilities. It represents the first step in the belief change towards empowerment in older adults and their initial attempt to embrace empowerment (Cleary and Zimmerman, 2004). Self-reflection helps older adults understand their roles in the family, community, and society, enabling them to make wiser decisions regarding the challenges they face.

##### 3.2.2.2. Overcoming barriers

Overcoming obstacles is one of the fundamental attributes of empowering older adults (van Corven et al., 2021), and it is widely applied in empowerment-related intervention programs. Kuo et al. (2014) defines empowerment for older adults as overcoming barriers within the social system, avoiding loss of income and welfare, gaining better resources, increasing job opportunities, and taking control of one’s own life. In an empowerment intervention program focused on healthcare for older adults (Sabouri et al., 2020), the first item in the program is overcoming obstacles. In fact, there may be a series of obstacles encountered in the process of empowering older adults, such as societal age discrimination, psychological concerns of older adults about their competency in work, and physiological cognitive decline. The key to overcoming these obstacles lies in social workers devising appropriate intervention measures to help older adults overcome the challenges they face.

##### 3.2.2.3. Taking responsibility

As the issue of aging becomes increasingly severe, older adults are gradually assuming the responsibility of self-care (Rudnicka et al., 2020), which is a crucial factor in the overall process of empowering older adults. A qualitative report (Yang and Zheng, 2017) highlights that older adults achieve empowerment by fulfilling various responsibilities, such as sharing household chores, participating in work, and engaging in social activities. Taking on these responsibilities not only alleviates the time, financial, and energy burdens on family caregivers but also signifies that empowerment goes beyond mere awareness and starts to manifest in the actions of older adults. Specifically, according to a study (Brieger et al., 2021), the significance of empowering older adults lies in their ability to express their own issues and needs, establish plans and goals, and effectively utilize their experiences and resources to achieve these objectives, such as acquiring knowledge, participating in work, and engaging in social activities.

#### 3.2.3. Behavioral autonomy

##### 3.2.3.1. Build connections

Building connections represents the initial stage of a transformation in the inner world and beliefs of older adults, which subsequently extends to the external world (Kamalpour et al., 2020). This transformation signifies a growing awareness of their needs and responsibilities, leading to proactive actions for change. They not only engage in more active communication with family and friends, fostering non-reciprocal friendships (Rodríguez-Medina et al., 2018), but they also begin to seek new connections. These connections include establishing positive relationships with others and the community, connecting with personal work, and engaging in collaboration and interaction with the human environment (Tsubouchi et al., 2021). This means that the process shifts from passively accepting external resources to actively engaging with them. Specifically, older adults begin to participate in social affairs in a formal way, making meaningful contributions to their families, communities, or society; which is an important principle of active aging (Chui et al., 2020).

##### 3.2.3.2. Active engagement

Active engagement is a critical component of empowering older adults. The key to successful empowerment lies in older adults being willing to take responsibility and participate in goal-setting and decision-making processes (Fotoukian et al., 2014). Several studies have shown that promoting the rights and well-being of older adults can be achieved through their involvement in relevant organizational activities and decision-making processes, which can enhance their social status and self-worth (Schepens et al., 2019). In terms of health, older adults who actively participate in medical decision-making and self-manage their health behaviors can improve treatment effectiveness (Yeh et al., 2018), and this can help them control the impact of diseases on their physical, emotional, and social well-being (Kayser et al., 2019). Similarly, Active participation in social and cultural activities contributes to the establishment of social support networks and enhances social engagement (Achmad, 2022). However, it is essential to note that when older adults are overly involved, it can pose challenges for healthcare professionals and community service providers, especially when their choices cannot be met, or conflicts arise between the parties involved.

##### 3.2.3.3. Self-determination

Self-determination is frequently mentioned in conceptual analyses and systematic reviews related to empowerment. It is considered the foundational empowerment theory, guiding the attributes in the empowerment process (Wu D. et al., 2021). Self-determination refers to the ability of older adults to identify their own needs and actively participate in significant processes (Wåhlin, 2017). It reflects the sense of choice individuals have in initiating and regulating actions. Setting specific goals and actively, autonomously, and self-regulated working towards achieving them are indicators of self-determination (Kalfoss, 2010).

### 3.3. Cases

“Cases” refers to the attributes defined when representing a concept. In this context, the proposed cases need to include the defined attributes and be accompanied by conceptual theories, providing specific practical examples, also known as model cases. These cases are adapted from literature or constructed by the authors.

#### 3.3.1. Model cases

Mr. W is a 67-year-old older adult with chronic health problems. After he was diagnosed with diabetes and high blood pressure in the hospital, Mr. W began to feel increasingly helpless. Then he listened to his family’s suggestions and began to attend community health lectures, learn how to control blood sugar and blood pressure and learn about some social resources, such as community hospitals and associations for the elderly. To that end, he also bought a smartphone and learned more about the disease and self-management through the web and social media. These resources made Mr. W realize that he needed to be more active in managing his illness, and he began to reflect on his lifestyle and eating habits, work with his doctor to develop his own life and treatment plan, and learn how to cope with the frustration and fear of his illness. With time, Mr. W’s illness gradually improved, and he also began to take the initiative to participate in social and cultural activities, such as community volunteers and school activities for elderly students. His social skills and access to information have also improved, and he has started using his smartphone and social media to stay in touch with family and friends and share his life and disease management experiences. Finally, through self-management and active participation, Mr. W has succeeded in empowering and improving his quality of life and happiness. His self-determined and proactive behavior allows him to feel more in control and autonomy, be a part of the community and contribute to society and his family.

### 3.4. Antecedents

The antecedent refers to the prerequisite events or attributes that must exist before a concept emerges. Therefore, in this conceptual analysis, the antecedents refer to the preceding factors that empower individuals in their later years. After carefully reading the literature and considering the complexity of empowering this population, we classify them into four categories: Physical obstacles, psychological concerns, personal needs, and external challenges. (1) Physical obstacles include: Declining physical function (Liu et al., 2019; Zhou, 2020; Qian et al., 2021), severity of diseases (Yang and Zeng, 2016; Liu and Tian, 2021; Shu et al., 2021), chronic illness (Zhou, 2020; Cheng et al., 2022), inadequate interpersonal communication skills (Ou, 2018; Zhan et al., 2018), and insomnia (Zheng, 2017; Yao, 2020). (2) Psychological concerns include: Aversion to becoming a burden (Cheng et al., 2022), high levels of anxiety (Li et al., 2017; Ren, 2020; Qi et al., 2021), disease-related shame (Zhang et al., 2021), past negative experiences (Zheng, 2017; Yang and Zeng, 2019; Xiao et al., 2022), and ineffective communication (Mi and Wu, 2023). (3) Personal needs include: Desire to overcome challenges (Hu and Zhu, 2022), thirst for knowledge acquisition (Zhang and Jiang, 2012), and willingness and effort to maintain personal well-being (Yao et al., 2016). Also, desire to take on family responsibilities (Zhang et al., 2015; Fan, 2020). (4) External challenges include: Limited financial resources (Dong, 2017; Li, 2018; Fan, 2020; Li et al., 2021), limited educational attainment (Chen et al., 2016; Fan, 2020; Yao et al., 2023), social exclusion (Fang et al., 2020; Guo et al., 2020; Hu and Zhu, 2022), and reduced social participation (Li et al., 2017; Yao, 2020).

### 3.5. Consequences

**Figure 3:**
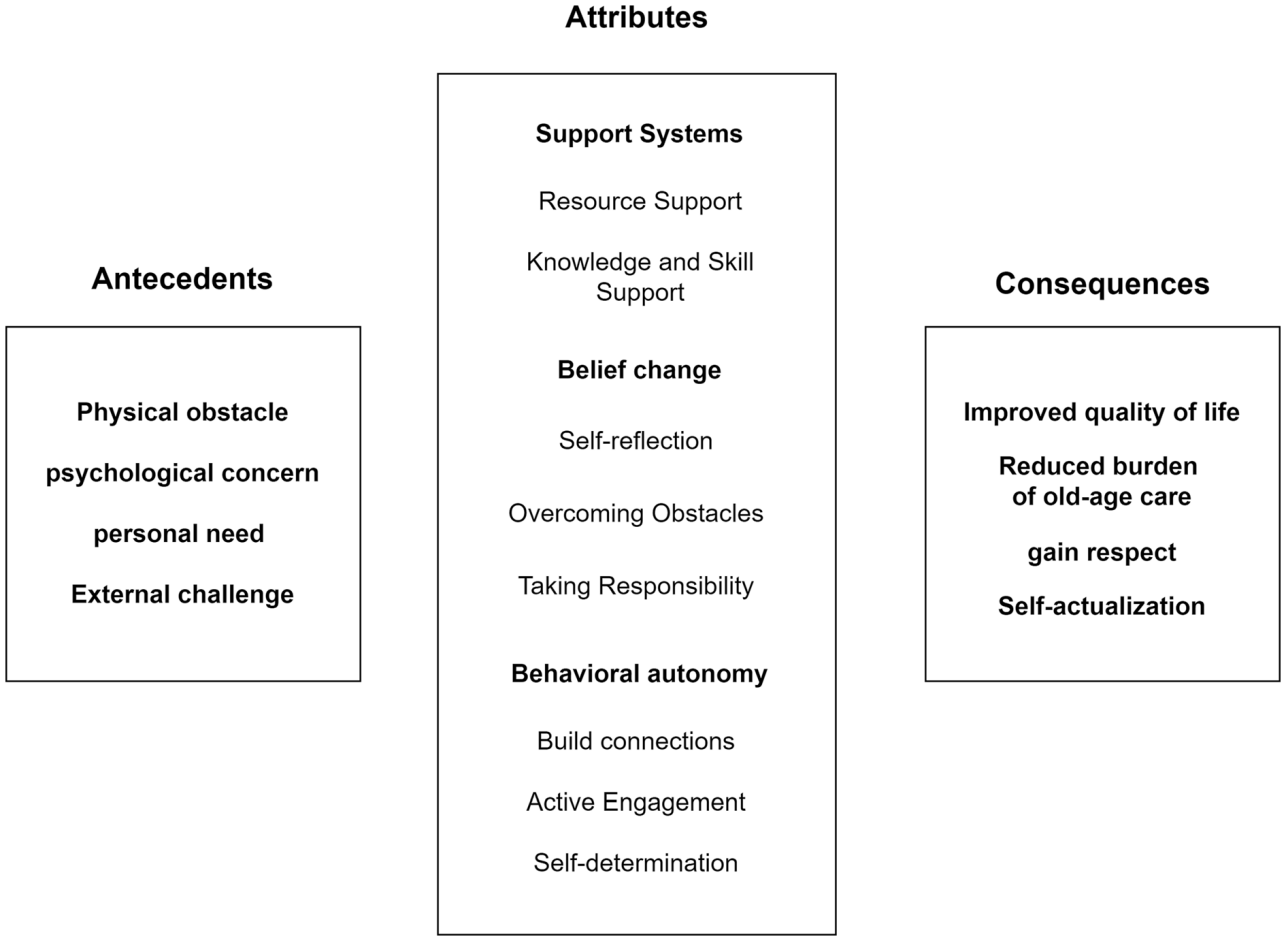
Proposed conceptual model of older empowerment in the China.

Consequence is an event or outcome that may occur due to the presence of a certain concept. According to the literature, when elderly individuals experience empowerment, there are four consequences: improved quality of life, reduced burden of old-age care, gain respect, and self-actualization of the older. Figure 3 illustrates the antecedents, attributes, and consequences of empowering older individuals within Chinese culture.

#### 3.5.1. Improved quality of life

One of the outcomes of empowering older individuals is the mitigation of diseases. With the transformation of their roles, healthcare providers offer older adults more health knowledge and collaborate in formulating treatment and care-related activities (Zhang and Jiang, 2012), paving the way for person-centered care. Consequently, older adults exhibit higher levels of trust in healthcare providers (Zhao et al., 2019), greater adherence to treatment plans (Wang et al., 2018; Qiu et al., 2021; Wu and Yang, 2021), and a greater willingness to actively engage in their own treatment and care processes (Chen et al., 2017), leading to improved health outcomes.

Another result of empowering older adults is the stabilization of their psychological well-being. Some studies indicate that during the empowerment process, older individuals begin to participate in more social activities (Dong, 2017; Zhao and Liang, 2017), engage in artistic endeavors (Yu et al., 2018; Feng and Yuan, 2020; Gao et al., 2021), and other practices that significantly reduce feelings of loneliness (Fang et al., 2020), depression (Du, 2018; Kang et al., 2020; Liu, 2020), fatigue (Li et al., 2017), and anxiety (Zhao et al., 2019). In turn, they develop expectations for a brighter future life (Zhou et al., 2021). Common evaluation indicators include self-efficacy, loneliness, satisfaction, and quality of life.

#### 3.5.2. Reduced burden of old-age care

With the improvement of personal capabilities among older individuals, they can better self-manage and reduce excessive reliance on their families and society. On the one hand, enhancing self-care abilities among older adults alleviates the burden on family caregivers. A study indicates that caregivers commonly experience reduced stress levels after empowering older adults (Zhang et al., 2021). On the other hand, offering self-care skills training for the elderly can alleviate the time and energy burden on caregivers (Liu, 2020). Furthermore, after successful empowerment, some older adults take on specific social or familial responsibilities, such as sharing household chores (Ma et al., 2018; Liu, 2020; Kong, 2021) or participating in work (Ren, 2020). Therefore, evaluation indicators for older adults’ self-management abilities include caregiver burden, self-care capabilities, and independence.

#### 3.5.3. Gain respect

The senior population often experiences a sense of being marginalized within society. However, by implementing a series of empowering measures, such as enhancing their knowledge and skills, establishing social networks, and improving interpersonal relationships, it may be possible for older individuals to integrate more effectively into society, thus alleviating their feelings of social exclusion (Fan, 2020). Actively participating in social activities enhances society’s awareness of the senior demographic, transforms negative perceptions and discriminatory practices, and gradually garners recognition (Zhu, 2019; Wang and Xu, 2022) and esteem (Liu, 2020; Han, 2021). Common evaluation indicators include social engagement, a sense of social identity, and a level of respect.

#### 3.5.4. Self-actualization

Self-actualization is the process through which individuals fulfill their self-worth and unleash their potential while striving to satisfy their highest needs (D’Souza and Gurin, 2016). On the other hand, the empowerment of older individuals involves granting them more choices and decision-making power, allowing them to age with confidence, autonomy, and dignity. Several studies have revealed that seniors engage in proactive social participation and establish interactive relationships with others; they gradually pursue self-worth, yearn for affirmation (Feng et al., 2019), and experience a sense of accomplishment (Wang, 2021). Specific evaluation indicators include a sense of achievement, self-worth, and self-fulfillment.

## 4. Discussion

In this conceptual analysis, we utilized Rodgers’ evolutionary concept analysis method to pinpoint the characteristics, cases, attributes, antecedents, and consequences of empowering elderly individuals in China. This in-depth analysis provides a clearer and more comprehensive understanding of the concept of empowerment among the elderly population in China. Based on our findings, we propose the following definition of elderly empowerment in China: In the context of interpersonal connections, empowering older individuals within Chinese culture can be defined as a series of processes that involve stimulating self-reflection through external resources such as emotional support, information, and collaborative activities. After reflecting on their current circumstances, older individuals overcome existing barriers and take proactive actions based on their decisions, leveraging their strengths in new environments.

Following Rodgers’ conceptual analysis approach, we have identified the primary elements of older empowerment within Chinese culture: support system, belief change, and behavioral autonomy. Further use of case studies helps to understand the concept of the presence or absence of attributes applied by workers in empowering older people. Some of our findings are consistent with other research on the empowerment of older adults (Fotoukian et al., 2014; Bravo et al., 2015). Supportive resources and proactive behaviors are commonly mentioned attributes in the study of older people’s empowerment in general and in Chinese culture. Among the attributes of belief change, self-reflection and overcoming obstacles are popular attributes of older people’s empowerment; however, we found that previous studies in other countries, mentioned in the above, did not mention the aspect of elderly individuals taking on family responsibilities. For example, in a conceptual analysis of empowerment in elderly individuals from Japanese culture (Tsubouchi et al., 2021), the attributes of empowerment included: self-interpretation, self-objectification and problem awareness, gathering necessary information and engaging in self-care behaviors, hopeful future based on decision-making, practicing proactive behavior and interaction with others and the community. This may be related to the emphasis on family ties in Chinese traditional culture (Lv et al., 2021). Most elderly individuals place their hopes in their families. This reflects their values, which include sharing housework, taking care of loved ones, and not burdening the family. As a result, taking on family responsibilities becomes an important factor in changing the beliefs of elderly individuals in China.

Our research indicates that the prerequisites for empowering elderly individuals include physical obstacles, psychological concerns, personal needs, and external challenges. In the context of empowering elderly individuals in China, addressing external challenges appears to be relatively more manageable compared to other factors. This suggests that, on one hand, elderly service providers in China should offer appropriate external resources and support (such as educational and training opportunities, social and cultural activities, healthcare services, etc.) to help older adults navigate challenges stemming from external circumstances (such as limited educational opportunities, decreased social participation, social exclusion, etc.; Kamalpour et al., 2020). On the other hand, efforts should be made to help older adults regain or enhance their social interaction skills. A study has confirmed the importance of peer assistance in the development of social skills among vulnerable populations (Rodríguez-Medina et al., 2016). Therefore, establishing companionships with older individuals and encouraging their active participation in social activities can be beneficial in addressing the external challenge of “reduced social engagement.

Upon examining the findings of the included studies, this concept analysis identified secondary changes and benefits that empowering older individual brings, including improved quality of life, reduced burden of old-age care, access to continuous support, and self-actualization of the older. This study emphasizes self-actualization as an outcome of empowering older individuals. Healthcare providers should view older individuals as complete individuals with social roles, no longer solely focused on recovering or alleviating their illnesses. Therefore, healthcare professionals can provide dignified, person-centered care, assist in nurturing the knowledge and skills of older individuals, and promote personal growth. Older individuals who aspire to self-actualization will prioritize pursuing personal value and meaning, fully unleashing their potential, contributing to society, and attaining self-fulfillment and a sense of accomplishment (Drabe et al., 2015). Recent research has reported the impact of empowerment on health, psychological well-being, behavior, sense of achievement, and happiness-related outcomes, which is consistent with our findings (Tsubouchi et al., 2021).

This study compared our research with studies on elderly empowerment in other countries and found that cultural factors have a relatively small impact on elderly empowerment. This finding aligns with previous research (Tsubouchi et al., 2021), but we should also consider the diverse lifestyles and health behaviors among elderly individuals in different time periods. We discovered that assuming responsibility may be a significant internal factor that drives elderly empowerment in China. Moreover, external resources, such as expectations and suggestions from family, friends, or professionals, are more likely to ignite the desire for empowerment among elderly individuals (Wu S.-Y. et al., 2021). To promote the empowerment of elderly individuals in Chinese culture, establishing group organizations that provide support resources and encourage them to take on specific responsibilities is meaningful (Fang et al., 2020).

This study indicates that elderly individuals who previously experienced passive problem-solving and limited opportunities to acquire resources are now able to take a more proactive approach to life with the help of external support in the context of empowerment. Moreover, older adults with higher levels of empowerment demonstrate increased interest in participating in various activities and possess the knowledge to make informed choices in order to achieve their desired goals (Longtin et al., 2010). Utilizing the identified antecedents in this study to provide services for the elderly is expected to increase their empowerment levels. We recommend the development of measurement tools and intervention plans tailored to Chinese elderly individuals, which reflect the empowerment attributes identified in this study. The significance of this research lies in the potential for a unified definition and concept to contribute to the development of elderly empowerment in China. Additionally, based on the antecedents identified in this study, suitable external resources can be further established to mitigate negative factors affecting the elderly.

## 5. Limitation

This study has a few limitations. Firstly, our database only includes mainstream databases in China, so it would be beneficial to incorporate relevant articles from other sources. Secondly, since this study did not differentiate based on gender among the elderly, it would be worthwhile to further investigate whether there are differences in the understanding and perception of empowerment among elderly individuals of different genders in future research.

## 6. Conclusion

Empowering older individuals is a complex concept influenced by direct economic, political, social, historical, and cultural contexts. It is a dynamic and evolving process that requires personal desire and effort, practical education, and support from available resources. It gives older individuals a sense of control, alleviates burdens for healthcare providers and caregivers, and improves community health outcomes. The defined concept, attributes, antecedents, consequences, and experiential references of empowering older individuals identified in this study can assess empowerment capacity in healthcare settings and inform theory-based interventions for empowering older individuals. This concept analysis provides valuable information for nursing practice, education, and research.

## Ethics statement

Written informed consent was obtained from the individual (s) for the publication of any potentially identifiable images or data included in this article.

## Author contributions

SBZ: Contributed to the design of the study. JFL: Contributed to the design of the study. JJZ: Conducted literature research, Article selection, Data extraction, Created the first draft of the manuscript. YTA: Conducted literature research, Article selection, Data extraction, Created the first draft of the manuscript. SQQ: Conducted literature research, Article selection, Data extraction, Created the first draft of the manuscript. XXX: Conducted literature research, Article selection, Data extraction, Created the first draft of the manuscript. HH: Reviewed and edited the manuscript. YW: Reviewed and edited the manuscript.
