# Supplementary material for: Empowerment of the older adults in the context of Chinese culture: an evolutionary concept analysis
Source: Front Psychol. 2023 Nov 9;14:1271315. doi: 10.3389/fpsyg.2023.1271315 (PMC10666161; doi:10.3389/fpsyg.2023.1271315)
Supplement: Supplementary file 2 [file Table_2.DOCX]

**Search strategies for all database**

| **Database** | **Search strategy** | |
| --- | --- | --- |
| **Pubmed** | #1 | "China"[MeSH Terms] |
|  | #2 | "Chinese"[Title/Abstract]) |
|  | #3 | #1 or #2 |
|  | #4 | "Aged"[MeSH Terms] OR "Aged, 80 and over"[MeSH Terms] |
|  | #5 | ("aged"[Title/Abstract] OR "elder people"[Title/Abstract] OR "elderly person"[Title/Abstract] OR "elderly patient"[Title/Abstract] OR "older people"[Title/Abstract] OR "older adults"[Title/Abstract] OR "old person"[Title/Abstract] OR "the aged"[Title/Abstract] OR "senior citizen"[Title/Abstract] |
|  | #6 | #4 or #5 |
|  | #7 | #3 AND #6 |
|  | #8 | "empowerment"[Title/Abstract] OR "patient empowerment"[Title/Abstract] OR "empower"[Title/Abstract] OR "disempower"[Title/Abstract] OR "enable"[Title/Abstract] OR "activation"[Title/Abstract] OR "engagement"[Title/Abstract] OR " collaboration"[Title/Abstract] OR "participation"[Title/Abstract] OR " support"[Title/Abstract] OR "rights"[Title/Abstract] OR "perceived control"[Title/Abstract] OR "Power"[Title/Abstract]) |
|  | #9 | #7 or #8 |
| **Web of Science** | #1 | AB=("China") OR AB=("Chinese") |
|  | #2 | ((((((((AB=(aged)) OR AB=(elder people)) OR AB=(elderly person)) OR AB=(elderly patient)) OR AB=(older people)) OR AB=(older adults)) OR AB=(older adults)) OR AB=(old person)) OR AB=(the aged) OR AB=(senior citizen) |
|  | #3 | #1 AND #2 |
|  | #4 | ((((((((AB=("empowerment")) OR AB=("patient empowerment")) OR AB=("empower")) OR AB=("disempower")) OR AB=("enable")) OR AB=("activation")) OR AB=("collaboration")) OR AB=("participation")) OR AB=("involvement")) OR AB=("support")) OR AB=("rights")) OR AB=("engagement")) OR AB=("perceived control")) OR AB=("Power") |
|  | #5 | #3 OR #4 |
| **China National Knowledge Infrastructure** | #1 | (TKA='老年'+'老年人'+'老者') AND (FT='赋权'+'赋能'+'失权'+'权利') AND (FT='中国'+'我国'+'本国'+'国内'+’国家’) |
| **Wangfang** | #1 | (摘要=老年人 or 主题=老人) OR (摘要=赋权 or主题=赋能) OR （(摘要=中国 or主题=我国) |
| **VIP** **Information Chinese Journal Service Platform** | #1 | (M=老年 OR 老年人 OR 老者 ) AND (U=赋权 OR 赋能 OR 失权 OR 权利 ) AND (U=中国 OR 我国 OR 本国 OR 国内 OR 国家) |
