# Supplementary material for: Empowerment of the older adults in the context of Chinese culture: an evolutionary concept analysis
Source: Front Psychol. 2023 Nov 9;14:1271315. doi: 10.3389/fpsyg.2023.1271315 (PMC10666161; doi:10.3389/fpsyg.2023.1271315)
Supplement: Supplementary file 3 [file Table_3.DOCX]

Table 2. The conceptual analysis includes the characteristics, antecedents, attributes, and consequences of the empowerment of the elderly in China as defined in the study.

| Author (year) | design | discipline | [sample](javascript:;) | antecedents | attributes | consequences |
| --- | --- | --- | --- | --- | --- | --- |
| (Chen et al., 2016) | quantitative study | Nursing | 220 | Knowledge, Technology, goodwill, education | Faith, self-management, personal resources, self-determination | A decline in self-management and care burden |
| (Chen et al., 2017) | quantitative study | Nursing | 136 | Disease status, gender, lifestyle | Health education, self-experience, active behavior | Quality of life and degree of disease remission |
| (Cheng et al., 2022) | quantitative study | Nursing | 101 | Burden of self | Reflection, self-management, and overcoming obstacles | The degree of burden decreased and the disease was relieved |
| (Du, 2018) | Mixed methods research | Nursing | 80 | Gender, amount of medical insurance, personal motivation, desire to take responsibility, duration of illness, and income | Disease remission and self-management level | Quality of life, confidence, self-efficacy, activities of living |
| (Fan, 2020) | Mixed methods research | Nursing | 360 | Age, gender, income, education, medical insurance, course of disease, mood | Peer support, positive coping, treatment confidence, and social support | Quality of life and partner support |
| (Feng et al., 2019) | review | Public health |  | Insufficient support resources and low level of knowledge | External support system, enhanced resource support and emotional support | Actively participate in social activities, improve the quality of life, and realize self-worth |
| (Gao et al., 2021) | quantitative study | Nursing | 210 | Disease cycle, disease knowledge | Self-management, belief in responsibility, and getting support | Emotional stability and self-reconstruction |
| (Guo et al., 2020) | quantitative study | Medicine | 94 | General information, type of disease, and amount of medical insurance | To stimulate self-awareness, assess the root causes of problems, take care of oneself, and participate in disease management | To improve the quality of life and stimulate internal potential |
| (Han, 2021) | Mixed methods research | Nursing | 770 | Gender, education level, insurance, disease status, health literacy | Psychological resilience, resource utilization, family support, self-responsibility, and social strength | Improved quality of life |
| (Kang et al., 2020) | quantitative study | Nursing | 174 | Disease status, gender | Define problems, express concerns, and offer help | Recovery from illness, motivation to participate in training, confidence, identity |
| (Kong, 2021) | quantitative study | Nursing | 60 | Anxiety, low motivation and unpleasant treatment experience | Overcome feelings of powerlessness, provide disease knowledge, self-reflect on problems, and encourage seeking social support | Reduce depression, anxiety, improve self-efficacy and quality of life |
| (Li et al., 2017) | quantitative study | Nursing | 220 | Social care resources are seriously insufficient, and the ability to live daily is low | Explore their own potential, actively participate in health care decision-making, improve self-decision-making ability | Gain confidence, comfort and safety in overcoming the disease and improve treatment compliance |
| (Li, 2018) | Mixed methods research | Nursing | 185 | Gender, family economics, Education level, | Medical resources, organizational management, social support | Improved quality of life |
| (Liu, 2020) | Mixed methods research | Nursing | 398 | Ethnicity, gender, place of residence, knowledge level, health literacy | Social support, perceived control, decisions about one's own health | improved quality of life |
| (Liu and Tian, 2021) | quantitative study | Nursing | 80 | Abnormal liver function, education level, alexithymia, longer course of disease | Self-awareness and self-decision-making, correcting bad behavior | Relationships, Remission of disease |
| (Ma et al., 2018) | quantitative study | Nursing | 80 | Health care provider needs survey | Rehabilitation knowledge, partnership, proactive training, self-conscious behavior change | Rehabilitation indicators changed, self-care ability increased, and the quality and satisfaction of living with illness increased |
| (Ou, 2018) | Mixed methods research | Public health | 16 | Discomfort, gradual marginalization, lack of sense of value and achievement, weakening of community communication and interpersonal relationship | Building relationships, self-expression, the power of engagement, dialogue communities, | Enhancement of self-efficacy, self-esteem and self-confidence, re-empowerment of life meaning and value, enhancement of community interaction and interpersonal relationship, enhancement of community participation and expression rights, |
| (Qi et al., 2021) | quantitative study | Nursing | 66 | After invasive treatment, affected by high medical cost, lack of disease cognition, disease knowledge mastery, psychological resilience, lack of cognition and other factors, postoperative patients are prone to depression, anxiety and other adverse emotions | Express inner feelings, self-regulate emotions, self-manage life behavior | Discover the meaning and quality of life |
| (Qian et al., 2021) | quantitative study | Nursing | 80 | Low self-care awareness, lack of self-care knowledge and skills | Narrating the experience and feelings of illness, daily nursing and disease knowledge education, expanding interpersonal communication experience | Self-care responsibility, self-concept, self-care skills |
| (Qiu et al., 2021) | quantitative study | Nursing | 69 | Low level of education, weak self-control, low compliance | Rehabilitation skills training, expand social circle, cultivate self-management consciousness, activate patients’ vitality | Mental health, emotional function, physiological function, physiological function |
| (Ren, 2020) | Mixed methods research | Medicine | 233 | Anxiety, submission, avoidance, patient monthly income, length of illness, place of residence, and number of children | Responsibility belief, knowledge and ability, action and performance, support and resources | Psychological state to obtain stability, self-esteem, satisfaction |
| (Shu et al., 2021) | quantitative study | Nursing | 224 | Lack of disease knowledge, too much pressure, family economic level | Problem reflection, self-management, emotional expression | Activities of daily living, active disease management, perceptual empowerment |
| (Wang, 2021) | review | Public health |  | Self-identity disorder, the dilemma of information shackles, narrow field of participation, | Social resource support system, create an emotional support atmosphere | Improve individual psychological control ability, improve individual practical control ability |
| (Wu and Yang, 2021) | quantitative study | Nursing | 90 | Gender, age, years of education, type of disease, complications | Knowledge education, expressing emotions, clarifying problems | Role adaptation, emotional processing, patient satisfaction, and balance ability |
| (Xiao et al., 2022) | quantitative study | Nursing | 187 | Age, complications, cardiac function classification, and self-care ability | Patient support, medical decision-making participation, role recognition, social support, doctor-patient relationship handling ability, and disease control confidence | Good cooperative relationship, self-control, quality of life, compliance |
| (Yang and Zeng, 2016) | review | Nursing |  |  | Beliefs and attitudes, knowledge and abilities, actions and performance, support and resources |  |
| (Yang and Zeng, 2019) | quantitative study | Nursing | 414 | Place of residence, education level, cohabitant, monthly income, proportion of medical insurance reimbursement | Belief in responsibility, getting support, increasing knowledge, participating in treatment, and rebuilding oneself | Self-satisfaction, self-acceptance, self-care, |
| (Yang and Zheng, 2017) | qualitative study | Nursing | 22 | Support from family members, support from friends, community support, health knowledge and skills | Daily life management, disease treatment and treatment management, knowledge acquisition management, resource utilization management | Try to stimulate the inner faith of elderly patients, full of the power of the social environment, empower others while getting self-empowerment |
| (Yao et al., 2016) | qualitative study | Public health | 812 | Ethnicity, prevalence of chronic diseases, participation in health education activities | Internal confidence, control and ability to solve problems independently | Self-management initiative |
| (Yao, 2020) | Mixed methods research | Nursing | 116 | Depressed, Anxiety, sleep quality, insomnia severity, behavioral disorders, and low social participation | Self-development, self-satisfaction, self-efficacy, self-awareness, building relationships, gaining trust and cooperation, managing and controlling illness and life | Decreased negative emotions, improved cognitive function, improved quality of life, gained respect, satisfied social interactions, and self-actualization |
| (Yu et al., 2018) | qualitative study | Nursing | 407 | Alexandria, functional somatic discomfort | Self-control, self-efficacy, problem solving, emotional adjustment, stress management, social support, self-motivated participation in decision making | The improvement of living habits, the expansion of friends, and the stability of mental state |
| (Zhang et al., 2015) | review | Nursing |  |  | Self-strength, stimulating responsibility, fulfilling health responsibility, self, family, society interaction | Achieving health |
| (Zhang et al., 2021) | qualitative study | Nursing | 274 | Education level, monthly income, physical examination times, admission time | Information technology resources, knowledge education, peer power, self-disclosure | Improved participation in treatment and increased adherence |
| (Zhang and Jiang, 2012) | review | Nursing |  | Feelings of shame about the diagnosis, desire to learn, and living in a shadow of fear | Unleash your potential and rebuild yourself | Accept the reality of the disease, self-change, manage the disease, and gain a sense of control |
| (Zhou et al., 2021) | qualitative study | Nursing | 188 | Health literacy, age, education level, course of disease, and income | Active decision making, active participation, responsibility belief, increasing knowledge | Rebuilding self and improving quality of life |
| (Zhou, 2020) | Mixed methods research | Medicine | 399 | Types of diseases, life experiences, attitudes to diseases, and needs for prevention and treatment | The ability to control one's own life and to integrate and influence one's environment, interpersonal relationships, Emotional support, external supervision, self-learning, knowledge and skills | Activate potential, expectations for future life, reduce inferiority, and gain respect |
| (Zhu, 2019) | review | Public health |  | Economic expenditure situation, social dependency, reduced status | Attitudes, values and beliefs; To confirm through collective experience; Knowledge and skills of critical thinking and action; Action | self-independence |
| (Dong, 2017) | Mixed methods research | Public health | 256 | Financial reasons, care burden, incomplete medical system, monotonous mental life, depression, low self-esteem | Collect data, build professional relationships, self-reflect, review life experiences, divert attention, rebuild confidence, organize activities, and stabilize work | The ability to access and control resources to one's advantage, self-control and social communication skills, To participate in the daily affairs of the society, by enhancing self-ability, exercise self-rights, to achieve the purpose of improving the client's own living state and living environment |
| (Zheng, 2017) | Mixed methods research | Nursing | 208 | Lack of knowledge of the disease, inability to cope with an emergency, poor sleep, anxiety the wrong medication | Guidance, assistance, supervision, information feedback, fulfilling the responsibility of health, social power, self-power | Self-efficacy, medical service utilization, disease rehabilitation, self-care ability |
| (Wang and Xu, 2022) | qualitative study | Nursing | 60 | Gender, disease type, education level |  | Self-efficacy, disease rehabilitation status, satisfaction and quality of life |
| (Yao et al., 2023) | qualitative study | Nursing | 100 | Gender, age, Education background, family relationship, | Clarifying problems, expressing feelings, health guidance, and co-management | The burden of care is reduced, and the disease is relieved |
| (Mi and Wu, 2023) | qualitative study | Nursing | 90 | Illness affects, communication is limited | Disease knowledge and skills training | Increased quality of life, increased patient satisfaction |
| (Hu and Zhu, 2022) | qualitative study | Public health | 501 | social pressure | Personal content, interpersonal relations, political society | Satisfaction, learning outcomes, social support |
| (Liu et al., 2019) | qualitative study | Nursing | 176 | Increased morbidity, disease risk, coverage, frailty, self-efficacy | Belief in responsibility, level of support, knowledge and skills, participation in therapy, rebuilding self | Social sense of belonging |
| (Wang et al., 2018) | qualitative study | Nursing | 94 | Gender, age, duration of illness, education level | The continuity of inner strength, the interaction between people and the environment, the constant renewal of self, and the mobilization of inner resources | Remission of disease, quality of life |
| (Zhao and Liang, 2017) | qualitative study | Nursing | 80 |  | Assessment, family support, community support, health education, empowerment management | Remission, relationships |
| (Zhan et al., 2018) | qualitative study | Nursing | 160 | Gender, age, course of disease, physical status | Nurse-patient relationship, building confidence in overcoming disease, establishing equal relationship, supervision | Mental health, emotional function, social function, health level, pain, physiological function |
| (Zhao et al., 2019) | qualitative study | Nursing | 122 | Catheter type, anxiety | Strengthen education, WeChat platform education, prevention of adverse events | Incidence of adverse events, physical life, social function, mental state, physical function |
| (Feng and Yuan, 2020) | qualitative study | Medicine | 219 | Inability to adhere to medication, lack of information and knowledge, disease recurrence | Health information, self-decision making, individuation, self-management, self-efficacy | Quality of life, compliance |
| (Li et al., 2021) | qualitative study | Nursing | 102 | Decreased swallowing function, malnutrition | Identify problems, express emotions, set goals, make plans, and evaluate results | Adaptive role, resilience, emotional processing ability, community resource level, life satisfaction |
